# Molecular Recombination Junction for Vacuum-Deposited Perovskite/Silicon Two-Terminal Tandem Solar Cells

**DOI:** 10.1021/acsenergylett.5c00155

**Published:** 2025-03-17

**Authors:** Sofía Chozas-Barrientos, Abhyuday Paliwal, Federico Ventosinos, Cristina Roldán-Carmona, Lidón Gil-Escrig, Vladimir Held, Perrine Carroy, Delfina Muñoz, Henk J. Bolink

**Affiliations:** † Instituto de Ciencia Molecular, 16781 Universidad de Valencia, Calle Catedratico Jose Beltran 2, 46980 Paterna, Spain; ‡ Université Grenoble Alpes, CEA, Liten, Campus Ines, 50 avenue du Lac Léman, F-73375 Le Bourget-du-Lac, France

## Abstract

The use of commercial, Czochralski-grown silicon wafers as bottom cells in two-terminal perovskite/silicon tandem configurations often leads to defects in the top perovskite absorber due to their rough surfaces, featuring μm-sized pyramids and saw damages. Most recombination junctions in two-terminal tandem cells employ high conductive indium tin oxide which increases the effect of local shunts in the top cell by connecting them. We use Suns–*V*_OC_ with selective illumination and external quantum efficiency measurements to identify these shunts. Additionally, we show that a molecular recombination junction composed of an n-doped C_60_ layer and a p-doped conjugated arylamine layer alleviates the effect of the shunts in the top cell, which we attribute to the lower lateral conductivity of the organic layers. This enables us to prepare two-terminal tandem devices using fully evaporated top cells on Czochralski textured silicon heterojunction cells with *V*_OC_s of up to 1.84 V and efficiencies above 22%.

## Graphical abstract

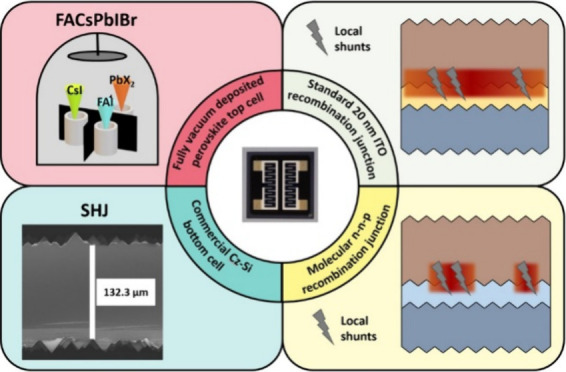

Photovoltaic (PV) energy has become one of the main contenders in achieving the 2050 Net Zero Emission goal, with silicon-based technologies dominating over 90% of the PV market.^1^ LONGi’s recent report of a new record efficiency of 27.30% for a silicon heterojunction cell,^2^ brings these technologies closer to their theoretical efficiency limit of 29.5%.^3,4^

In the quest for bringing the performance of silicon PV modules beyond this theoretical limit, perovskite/silicon tandem configurations have gained much interest. The incorporation of a wide bandgap perovskite semiconductor on top of a silicon bottom cell enables a broader and more efficient utilization of the incident solar light which results in improved power conversion efficiencies with respect to the single-junction counterparts. The theoretical efficiency limit of double-junction tandem solar cells ranges between 45 and 47%.^5–7^ Metal halide perovskites find their potential as top cells in the tunability of their bandgap, which enables them to efficiently complement the absorption characteristics of the bottom cell, their high absorption coefficient, and their low-temperature and low-cost processing. The certified record efficiency for perovskite/silicon tandem solar cells stands at 34.6% as reported by LONGi Green Energy Technology.^7^

These high efficiency tandem cells are typically monolithic or two-terminal configurations in which a p-i-n perovskite top cell is electrically connected in series to an n-type crystalline silicon heterojunction bottom cell through a recombination junction (RJ). The RJ is tasked with the recombination of the majority charge carriers collected at each side of the RJ. The most commonly used RJ material in the best performing tandem solar cells is indium tin oxide (ITO).^8–12^ However, the high lateral conductivity of ITO increases the detrimental effect of local shunts in the top cell.^13,14^ The occurrence of shunts is a common scenario when perovskite absorbers are deposited on rough surfaces such as that of industrial Czochralski-grown silicon wafers which besides the micrometer-sized pyramids also feature abrupt thickness changes caused by the sawing process of the ingots.^15^ Vacuum deposited perovskites are of interest for two-terminal (2T) perovskite/silicon tandem cells due to their conformal deposition over the textured surface of silicon cells.^16^ However, the conformal coating also implies that there is little to no smoothening of abrupt thickness changes on the silicon bottom cell that are frequently present due to saw damage or imperfect texturing. As a result, local shunt paths can form in the perovskite top cell. When instead front side polished silicon cells are employed as bottom cells in combination with solvent processed perovskite top cells, as is common in high efficiency perovskite/silicon tandem reports,^17–25^ these shunts are mostly prevented.

Here we present 2T perovskite/silicon tandem cells employing industrial, CZ fully textured silicon heterojunction (SHJ) bottom cells and fully vapor-deposited perovskite top cells. In it, we employ an all-organic recombination junction composed of an n-doped C_60_ layer and a p-doped organic conjugated arylamine (TaTm) layer. TaTm is N4,N4,N4″,N4″-tetra­([1,1′-biphenyl]-4-yl)-[1,1′:4′,1″-terphenyl]-4,4″-diamine. It is shown that the reduced lateral conductivity of these organic layers effectively alleviates shunting in the top cell compared to similar devices employing ITO recombination junctions. Furthermore, we employ a Suns–*V*_OC_ with selective illumination analysis that allows us to determine the independent *V*_OC_ of the silicon bottom cell within the 2T tandem cells. From this analysis we find that the silicon bottom cell can generate a *V*_OC_ of 0.62 V whereas the 2T tandem yields *V*_OC_s of up to 1.84 eV when featuring the all-organic RJ.

Figure 1a shows the overall stack of the tandem solar cells reported in this work. All the layers are fabricated via dry, vacuum deposition methods. The bottom cell is a rear-junction, SHJ cell. Details of the bottom cell stack can be found in the Supporting Information (SI). The SHJ bottom cells are obtained from M2 wafers that are laser-cut into 3 × 3 cm substrates. The original M2 wafers are characterized before cutting using a standard photoconductance decay technique referred to as a Sinton test and yield implied *V*_OC_s of up to 0.744 V and implied FFs up to 85.6%. After cutting the M2 wafers to 3 × 3 cm^2^ substrates the *V*_OC_s decreased to 0.7 V.

**Figure 1:**
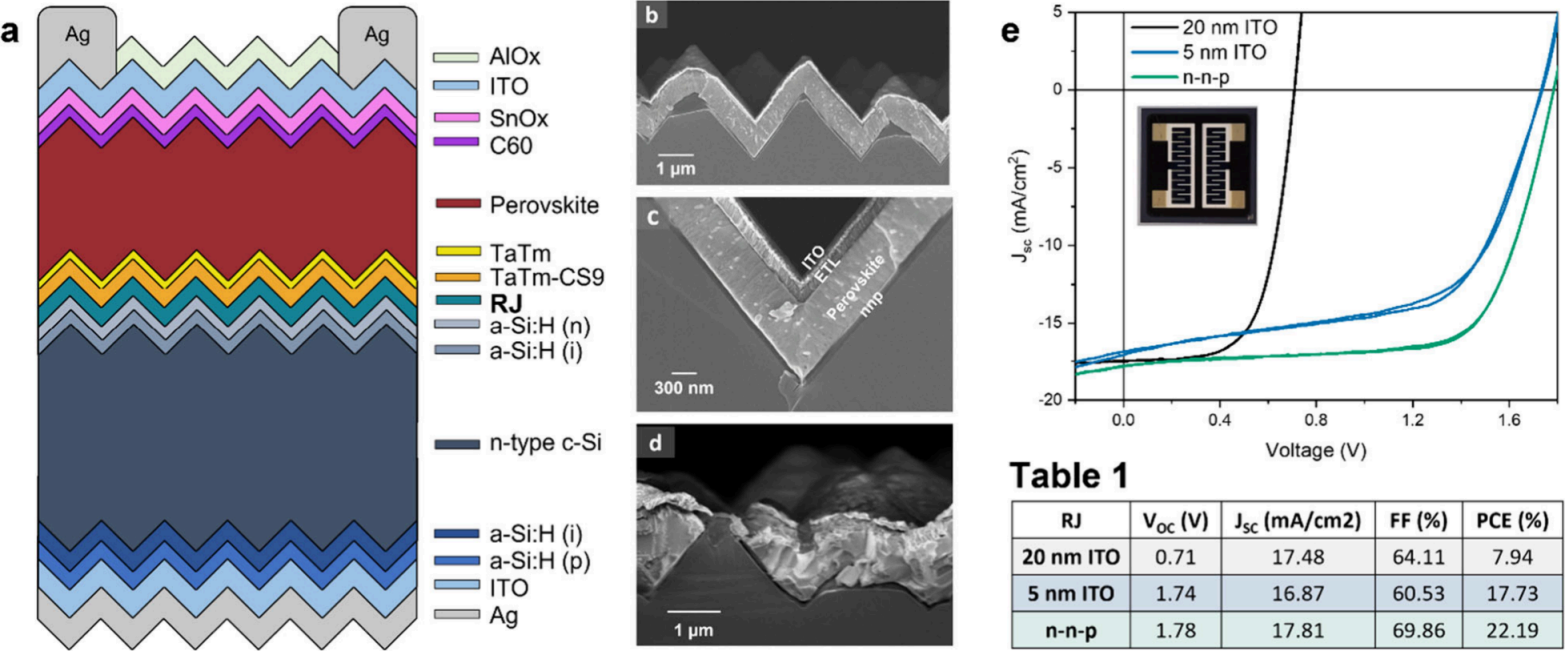
(a) Tandem stack explained in detail in the main text. (b) Conformally covered pyramids with the top cell consisting of the organic n–n–p RJ. (c) Conformal coverage of the valley of the pyramid without material accumulation. (d) Uncovered pyramid in a tandem with 20 nm ITO RJ, leading to a complete short of the tandem cell. (e) *J*–*V* characteristics of (black curve) tandem using the standard 20 nm thick ITO RJ, (blue curve) tandem using a 5 nm ultrathin ITO RJ, (green curve) tandem using our all-molecular n–n–p RJ. (Table 1) Photovoltaic parameters of tandems with 20 nm ITO RJ, 5 nm ITO RJ, and n–n–p RJ. Please see Figure S4 for the statistics of the *J*–*V* curves of the tandems with the three different RJ’s.

The SHJ cells are based on an inverted emitter configuration base on a n-type wafer, hence a p–i–n configuration, and thus the perovskite top cell must also be deposited in the p–i–n sequence such that electrons from the silicon cell can recombine with holes from the perovskite cell in the recombination junction (RJ). For our first trials a 20 nm ITO layer was deposited on top of the a-Si:H (n) layer as the RJ. The perovskite top cells prepared on top of the bottom silicon cell had the following stack sequence. The hole transporting layer (HTL) consists of a N4,N4,N4″,N4″-tetra­([1,1′-biphenyl]-4-yl)-[1,1′:4′,1″-terphenyl]-4,4″-diamine (TaTm) molecule that is partially oxidized by thermally cosubliming 2,2′-(perfluoronaphthalene-2,6-diylidene)­dimalononitrile (F6-TCNNQ) to form a p-type doped layer. On top of this p-doped TaTm layer a thin layer of undoped TaTm is deposited to separate it from the perovskite absorber as this reduces recombination at the perovskite–HTL interface.^26^ The composition of the top perovskite absorber was CsFAPbIBr, prepared via a three-source thermal coevaporation process. Our previously reported four-source protocol, using CsI, FAI, PbI_2_ and PbBr_2_ as precursors,^27^ was simplified by unifying the two latter into a mixed halide alloy Pb­(I_1–*n*_Br_*n*_)_2_. PbI_2_ and PbBr_2_ were added in stoichiometric proportions in a crucible and melted at 360 °C in N_2_ at atmospheric pressure.^28^ The mixed-cation, mixed-halide perovskite was then fabricated by the simultaneous sublimation of CsI, FAI, and Pb­(I_1–*n*_Br_*n*_)_2_ yielding a perovskite of general formula Cs_0.2_FA_0.8_Pb­(I_0.8_Br_0.2_)_3_ with a bandgap of 1.66 eV. The details of the experimental conditions are provided in the SI. as well as the absorbance and XRD spectra of the perovskite (Figures S1 and S2). After the perovskite, fullerene C_60_ was sublimed as the electron transporting layer (ETL) as it has a good electron mobility, and the lowest unoccupied molecular orbital (LUMO) aligns well with the conduction band of the selected perovskite composition. C_60_ was then covered by a layer of SnO_2_ deposited via atomic layer deposition (ALD) which facilitates charge extraction and serves as buffer layer to protect the underlaying stack.^29–31^ Indium tin oxide (ITO) used as transparent electrode and/or as RJ was deposited via pulsed laser deposition (PLD) in a soft process which effectively mitigates damage on the underlying stack as previously reported.^32,33^ An Ag grid with the layout presented in the inset in Figure 1e was then thermally evaporated on top of the front ITO electrode. The resulting tandem cells were finished with a low temperature ALD-deposited Al_2_O_3_ to protect them from environmental agents, since characterization is performed in air.^34^ To reduce the reflection of light a layer of LiF was thermally evaporated as an antireflection coating that works in conjuction with the AlOx layer. Figure 1b shows a cross-section SEM image where the pyramids of the silicon cell are clearly seen, as well as the conformally coated top perovskite cell. Figure 1c depicts a magnified cross-sectional SEM image in which the different layers of the top perovskite cell stack can be distinguished. To check the quality of the perovskite, a single-junction reference device was prepared on patterned ITO glass. The photovoltaic parameters of the perovskite reference can be found in Figure S3.

The black curve in Figure 1e shows the *J*–*V* characteristics under 1 sun illumination using a Wavelabs LED based sun simulator of a tandem solar cell using a 20 nm thick ITO RJ. Its performance resembles that of a filtered silicon bottom cell with current densities of about 18 mA/cm^2^ and *V*_OC_s close to 0.7 V, which indicates that the top cell is completely short circuited and does not provide any photocurrent to the tandem. Sahli et al. reported a similar behavior when using fully textured silicon bottom cells featuring the standard ITO RJ thickness and a thermally evaporated HTL, in this case Spiro-TTB.^35^ They attributed the shorting to a suboptimal ITO/HTL interface which led to the partial detachment of the organic HTL from the textured ITO covered bottom cells. For our stacks with the 20 nm ITO RJ, we are uncertain of the exact nature of the shunts. However, by evaluating many SEM images from many different batches we found one where in one case there was a suboptimal coverage of a pyramid on the bottom cell surface leading to an exposed pyramid tip as is shown in the cross-sectional SEM image in Figure 1d. Uncovered pyramids could explain the complete shunting of the perovskite top cell.

To reduce this shunting effect, we decided to use an n–p RJ by directly depositing the partially oxidized p-type TaTm layer on the n-type hydrogenated amorphous silicon layer (a-Si:H (n)). This is similar to the p–n junction Hyun et al. (2022) prepared in a 2T perovskite/TOPCon tandem through direct contact between the SnOx ETL of the top nip perovskite cell and the p-type silicon emitter of the bottom cell.^36^ However, somewhat to our surprise the implementation of this direct n–p junction in our tandem cells leads to extraction losses. As shown in Figure S5, the *J*–*V* characteristics under 1 sun illumination exhibit an S-shape typical of devices where a potential barrier is formed, which leads to inefficient charge extraction and hence to poor FFs and performances.^37,38^

We then prepared a different stack in which we deposited a partially reduced C_60_ (n-C_60_) on the a-Si:H (n) to ensure a good n–-n contact. The reduced C_60_ layer is obtained by thermally cosubliming C_60_ with the electron donor N1,N4-bis­(tri-*p*-tolylphosphoranylidene)­benzene-1,4-diamine (PhIm) at rates of 0.5 and 0.2 Å/s, respectively (more details can be found in the SI). Via Hall effect measurements we were able to demonstrate that this doped C_60_ is an n-type semiconductor (Figure S6). Following that n–n bilayer we then deposited the p-doped TaTm layer followed by the remaining layers of the previously described perovskite stack. Hence, these cells have a RJ consisting of an n–n–p junction.

Figure 1e, green line, shows a typical *J*–*V* curve (under 1 sun illumination) for the 2T tandem device with the n–n–p RJ. From this curve the key device performance parameters can be obtained. These are depicted in Table 1. The short circuit current density *J*_SC_ is 17.8 mA/cm^2^ which compares well with what was obtained from the silicon cell under 50 mW/cm^2^ illumination and with the results obtained from the 2T tandem cell with the 20 nm ITO RJ. Hence, from the *J*–*V* curve we can deduce that reasonable current matching is obtained (in the next section we display the EQE data for the two subcells confirming this). The open circuit voltage (*V*_OC_) is 1.78 V, rather low for a 2T tandem cell. However, *V*_OC_ values of up to 1.84 V were obtained using this n–n–p RJ as presented in Figure S7. Finally, the fill factor (FF) close to 70% is again somewhat low. As a result, the power conversion efficiency (PCE) of this type of tandem cell is only slightly above 22%. However, compared with the tandem cells employing the 20 nm ITO RJ, these values are significantly improved. We attribute this enhanced performance to the reduced lateral conductivity of the doped organic layers with respect to the 20 nm thick ITO RJ (8 × 10^–3^ S/cm). This is essential to reduce the effect of isolated shunt paths, leading to improved *V*_OC_s and FFs. To test if the lateral conductivity of the RJ is indeed the reason for the improved performance, we prepared another 2T tandem using an ITO RJ with a reduced thickness as has been previously demonstrated to work in the literature.^39–42^ For this tandem device a 5 nm thick ITO RJ was deposited on top of the silicon bottom cell with otherwise the same top cell architecture described above. The blue curve in Figure 1e shows the *J*–*V* characteristics under 1 sun illumination of the 2T tandem device with the 5 nm ITO RJ. When going from 20 to 5 nm thick ITO RJ, the sheet resistance of the film increases from 5E2 to 2E3 Ω/sq, respectively, as estimated from previous work done in our group,^43^ which results in an improved *V*_OC_ and overall performance of the device. Nonetheless, the tandem devices featuring the n–n–p junction still present a higher shunt resistance as can be seen by the lower slope of the *J*–*V* curve close to *J*_SC_ and by the dark *J*–*V* curves (Figure S8). We note that the FF of the cells using the ITO RJ of 5 nm is lower than that of the cells using 20 nm ITO RJ. The cells with the 20 nm ITO RJ are essentially functioning as optically filtered silicon cells and thus the FF is for a single junction cell, whereas the cells with a 5 nm ITO RJ do function as tandem cells (although poorly).

The reduction of shunts on the overall tandem device when using the n–n–p junction presented in this work was further revealed via external quantum efficiency (EQE) measurements. The EQE was measured using an Enlitech QE-R Quantum Efficiency System. To measure the spectral response of each subcell in the 300–1200 nm range we use a beam spot with an area of 1 mm^2^. A halogen lamp is used to illuminate the subcell for which the EQE is not being analyzed. When the EQE of the perovskite top cell was analyzed a 850 nm long pass filter was applied to the halogen lamp to saturate the current generated by the SHJ bottom cell, and when the EQE of the SHJ bottom cell was analyzed a 550 nm short pass filter was used to saturate the current generated by the perovskite top cell. The current mismatch generated within the tandem device by this light bias leads to a shift in the operating voltage of the subcell being measured. Hence, without a voltage bias the subcell under measurement is in reverse bias and therefore a bias voltage is needed to bring it back to short-circuit conditions.^44^ The voltage bias applied is in the range of the *V*_OC_ of the nonanalyzed subcell, that is around 0.6 V when analyzing the EQE of the perovskite top cell and around 1.0 V when analyzing the EQE of the SHJ bottom cell.

As presented in Figure 2a, when using the 5 nm thick ITO as RJ, a parasitic response is recorded for the perovskite for photons of energies below its bandgap (>760 nm). In the literature, such artifacts in the EQE profiles of multijunction photovoltaic devices have already been reported and are known to appear when the subcell being tested presents nonideal properties, mainly low shunt resistances.^45^ In fact, the parasitic signal recorded in this part of the spectrum corresponds to a simultaneous response of the bottom cell. Figure 2b gives a schematic representation of this measurement artifact. Part of the photocurrent generated in the bottom cell flows through the low shunt resistance of the top cell (red dashed line) such that a signal is recorded. Moreover, as a consequence of its low *R*_sh_, part of the current generated in the top cell may also preferentially flow through this path of lower resistance rather than the diode (blue dotted line), which leads to a lower spectral response in the visible part of the spectrum. However, when the 5 nm thick ITO RJ is replaced by the n-n-p junction the tandem cells no longer show this artifact in the EQE analysis. As can be seen from the solid black curve in Figure 2a, the EQE of the perovskite top cell drops to zero in the IR region of the spectrum. For more details regarding the voltage biasing, refer to Figure S10 of the SI.

**Figure 2:**
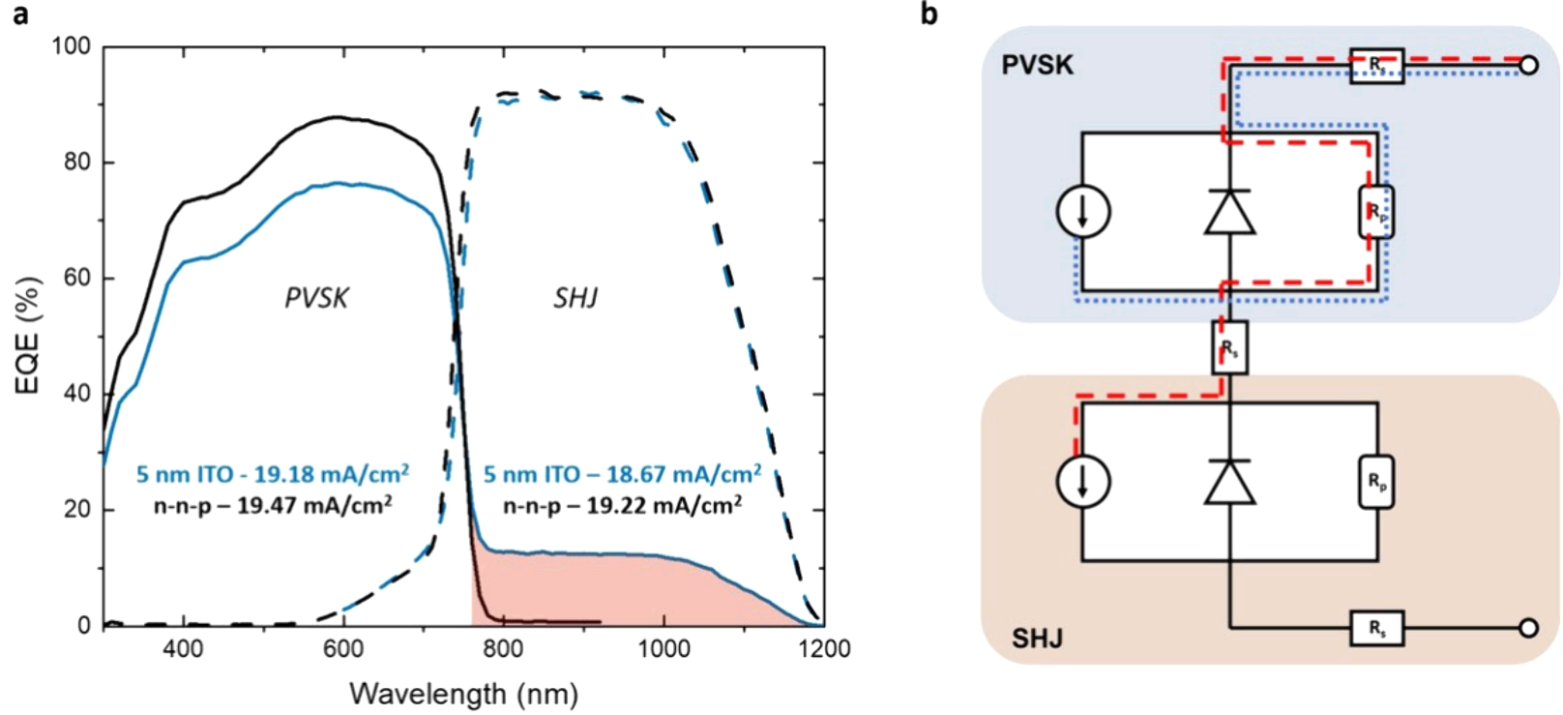
(a) EQE measurements for tandem devices employing n–n–p RJ (black) and 5 nm ITO RJ (blue). Shaded in red is shown the artifact measured due to shunts in the top cell. These curves are representative for a wide range of devices; see Figure S9 for a representation of multiple curves. (b) Proposed explanation of the artifacts seen in the EQE data. The dashed red line represents current generated in the bottom cell flowing through the low shunt resistance in the top cell which would give rise to a signal in the infrared part of the spectrum. The dotted blue line represents current generated in the top cell flowing through its low shunt resistance decreasing the signal in the visible part of the spectrum.

As shown in Figure 2a, the *J*_SC_ values integrated from the EQE appear to be higher than those obtained from JV measurements presented in Table 1. However, it should be considered that due to the 1 mm^2^ beam spot size, the value obtained from EQE does not take into account the shadowing effect of the gridline, as the beam spot impinges in the space between the metal fingers (Figure S11). Hence, this shadowing effect should be taken into account to properly compare the *J*_SC_ obtained from EQE to that obtained from JV measurements. In this regard, considering that the metal fingers represent 11% of the total area, a factor of 0.89 can be applied to the *J*_SC_ integrated from EQE measurements. When this shadowing factor is applied, the corrected *J*_SC_ values obtained from the EQE measurements for tandems with both ITO and n–n–p RJ (17.07 mA/cm^2^ and 17.32 mA/cm^2^, respectively) are in good agreement with those shown in Table 1 obtained from *J*–*V* measurements.

Although the *J*_SC_ values are obtained integrating the EQE over the whole spectrum (300–1200 nm), it should be noted that for the tandem with a thin ITO RJ it is composed of two separate contributions as stated above: the current properly generated in the top cell (300–780 nm) which accounts for 16.90 mA/cm^2^, and a leakage current generated in the bottom cell flowing through the shunt of the top cell (780–1200) which accounts for 2,13 mA/cm^2^. As explained by Oviedo et al. this can happen when the limiting subcell is heavily shunted, causing it to operate under reverse bias close to *J*_SC_.^46^

To further inspect and quantify the shunting characteristic of our devices, we performed what we call *Suns–V*_*OC*_ *with selective illumination*. Typically, the Suns–*V*_OC_ technique relies on measuring the voltage that develops in a photovoltaic device as a function of the intensity of illumination.^47^ This can be done by using several neutral density filters in order not to alter the spectrum of the utilized solar simulator. From the *V* vs flux data set, both the shunt resistance and the diode quality parameter can be extracted. In this type of experiment, since there is no current flow, no information on *R*_s_ can be obtained, which leads to pseudo-*J*–*V* curves which show the upper limit of the FF for an ideal *R*_s_. In this work we determine the *V*_OC_ as a function of illumination intensity but using two different light sources chosen such that they selectively excite only one of the two subcells in the tandem device. In order to excite only the top cell, we use a 450 nm blue laser with a maximum power of 130 mW, while to excite the silicon bottom subcell we use a 910 nm infrared laser of 200 mW of power. This type of measurement has been done previously to estimate the independent *V*_OC_ of each subcell for both micromorph and amorphous silicon-based tandem cells,^48,49^ and to study equivalent circuit models.^50^ However, as far as we know this is the first time it is applied to perovskite/silicon tandem cells. In Figure 3, the voltage as a function of flux is depicted for top and bottom subcells for both types of tandems, the one using the 5 nm thick ITO RJ and the one using the n–n–p junction as the RJ. When these cells are illuminated with the blue laser (which is mainly absorbed in the perovskite layer), a clear difference can be observed in the voltage versus flux curves. For the tandem cell that uses the 5 nm thick ITO RJ a somewhat linear behavior of the voltage on the flux is observed, whereas for those that use the n–n–p junction as the RJ, the voltage vs flux is best described by a logarithmic function. A statistical study was performed for a set of over 20 tandem cells obtained from many different deposition runs and is presented in Figure S12 of the SI. They all show the tendency described above. Moreover, this is correlated with dark JV measurements which further support the alleviation of shunts when the n–n–p RJ presented here is implemented.^51–53^

**Figure 3:**
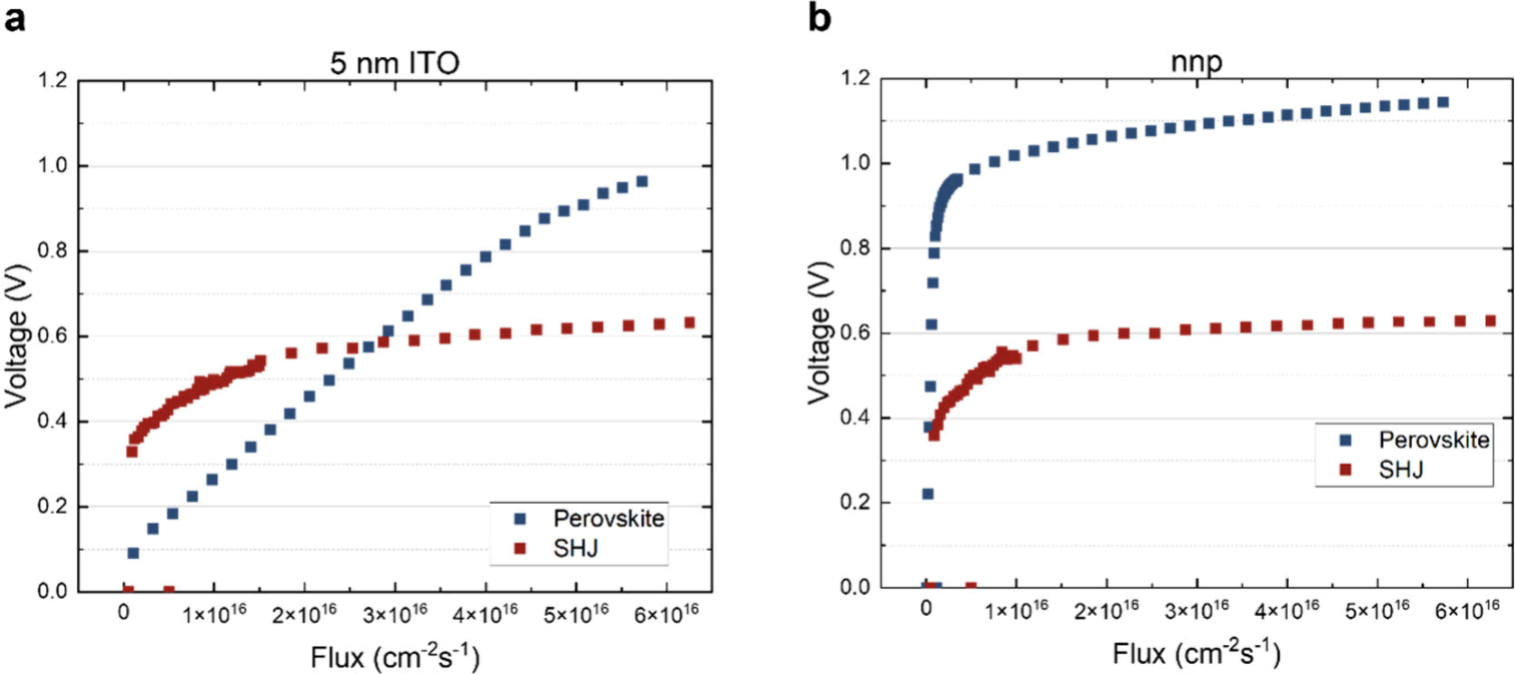
Suns–*V*_OC_ with selective illumination curves for both (a) 5 nm ITO RJ (left plot) and (b) the n–n–p RJ (right plot). The linear response of the perovskite for the device containing 5 nm ITO as RJ is an indication of the lower shunting resistance in the top cell, compared to the n–n–p RJ counterpart.

As the *V*_OC_ is proportional to the natural logarithm of *J*_SC_, which in turn is linear with intensity of illumination, a logarithmic behavior of voltage versus flux is to be expected. The linear behavior observed for the perovskite in the tandem using the ITO RJ points to shunting problems in this subcell, while a normal logarithmic behavior is seen in both silicon bottom cells.

Another interesting observation can be drawn from the voltage versus flux curves, namely the value the voltage reaches at high flux. In particular at the flux equivalent to 1 sun (that would lead to the *J*_SC_ of the subcell which can be obtained from the EQE analysis) the voltage obtained gives a good estimate of the open circuit voltage at 1 sun for each subcell. As discussed by Holovsky et al.,^48^ the accuracy of this estimation depends on the selectivity of the illumination chosen. For example, using the EQE data for the tandem employing the 5 nm ITO RJ and integrating it over the AM 1.5G spectrum we derive a *J*_SC_ of the bottom subcell of 18.5 mA/cm^2^. Dividing this value by the elementary charge *q* gives the amount of photocarriers generated. From the EQE at the wavelength of the infrared laser (910 nm in this case), we can derive the flux that is needed to generate the same number of photocarriers. This gives a flux of 4.5 × 10^16^ cm^–2^ s^–1^, which from Figure 3 corresponds to a voltage of *V*_OC _cSi_ = 0.61 V. This voltage is low for state-of-the-art SHJ cells. However, the cutting to 3 × 3 cm^2^ substrates, handling and the filtering by the perovskite top cell, can cause such a reduction compared to what was obtained from inferred *V*_OC_ on the M2 original wafers using the Sinton lifetime test.^54^ We apply this method only for the bottom cell, as for the perovskite top cell it is less easy. This is due to the fact that the perovskite has a nonzero photoluminescence when illuminated with the blue laser. A part of the re-emitted light will be absorbed by the silicon bottom cell, which will generate a voltage in the silicon that increases the recorded voltage, misleading the true voltage of the perovskite cell. That is why we propose to estimate the *V*_OC_ of the top cell by subtracting the *V*_OC_ of the bottom cell from the total *V*_OC_ of the device under 1 sun illumination: *V*_OC_top_ = *V*_OC_1sun_ – *V*_OC_cSi_.

Thus, we demonstrated that vacuum cosublimation of a mixed-cation, mixed-halide perovskite on textured CZ wafers leads to conformal coating and in general good coverage of the silicon textured bottom cells. The performance of the 2T perovskite/silicon tandems depends strongly on the type of charge recombination junction used. A typical 20 nm ITO layer leads to very poor performances, more similar to silicon cells with reduced current density due to the filtering effect of the perovskite top cell. Exchanging the ITO charge recombination junction for a bilayer of n- and p-doped organic electron and hole transporting molecules leads to strong improvements in the tandem cells, reaching *V*_OC_s of up to 1.84 V and power conversion efficiencies of 22%. We show that this improvement is primarily due to the increased resistance of this bilayer. These efficiencies are low when compared to those obtained from tandems prepared using solvent-assisted perovskite deposition methods and by using polished, flattened, or nanotextured silicon bottom cells. However, our results are relevant as they involve fully sublimed perovskite cells on industrial CZ-grown micrometer textured wafers. We demonstrate that EQE measurements can provide qualitative information about shunts in each subcell. Moreover, the Suns–*V*_OC_ with selective illumination technique applied in this work supports this qualitative information while also providing quantitative information on the voltage generated by each subcell within the tandem device. We hope that this work may encourage the scientific community to further test these easily accessible analysis techniques in high-efficiency tandem solar cells.

## Supplementary Material



## Notes

Detailed experimental conditions and characterization information, absorbance, XRD difractogram of the wide band gap Cs_0.2_FA_0.8_Pb­(I_0.8_Br_0.2_)_3_ perovskite, additional device configurations, Hall effect measurement of the doped C_60_ film, and device statistics (PDF)
