# Supplementary material for: Molecular Recombination Junction for Vacuum-Deposited Perovskite/Silicon Two-Terminal Tandem Solar Cells
Source: ACS Energy Lett. 2025 Mar 17;10(4):1733–40. doi: 10.1021/acsenergylett.5c00155 (PMC12127976; doi:10.1021/acsenergylett.5c00155)
Supplement: Supplementary file 1 [file nz5c00155_si_001.pdf]

SUPPORTING INFORMATION

Molecular Recombination Junction for Vacuum-Deposited Perovskite/Silicon 2-Terminal Tandem Solar Cells

*Sofía Chozas Barrientos[1], Abhyuday Paliwal[1], Federico Ventosinos[1], Cristina Roldán-Carmona[1], Lidón Gil-Escrig[1], Vladimir Held[1], Perrine Carroy[2], Delfina Muñoz[2], Henk J. Bolink[1] **

1) Instituto de Ciencia Molecular, Universidad de Valencia, Calle Catedratico Jose Beltran 2, 46980 Paterna, Spain

2) Université Grenoble Alpes, CEA, LITEN, Campus INES, 50 avenue du Lac Léman, F-73375 Le Bourget-du-Lac, France

Corresponding Author: Henk J. Bolink - henk.bolink@uv.es

Detailed experimental conditions:

Aside from ITO and SnO_2 layers that were deposited via PLD and ALD respectively, all other layers were deposited via thermal sublimation in high vacuum chambers. The high

vacuum chambers are placed inside N_2-filled gloveboxes with H_2O and O_2 levels < 0.1 ppm. For deposition, vacuum chambers are evacuated to a pressure of 10^{-6} mbar using turbomolecular pumps coupled to a scroll pump. The vacuum chambers feature four Creaphys temperature-controlled evaporation sources fitted with ceramic crucibles. All sources are directed upwards with a 90° angle with respect to the floor of the chamber. A distance of 20 cm separates the substrate holder from the sources. Three quartz crystal microbalance (QCM) sensors are employed to monitor the deposition rate of each source. Additionally, a QCM sensor is placed at substrate level to monitor the total deposition rate.

Thickness calibration of the organic charge transporting molecules (TaTm and C_{60}) and their dopants (F6-TCNNQ and PhIm) was carried out by individually subliming each material and then calculating a calibration factor comparing the thickness read on the QCM sensors with that measured using an Ambios XP1 profilometer.

For the p-TaTm layer TaTm and F6-TCNNQ were co-sublimed at a rate of 0.8 and 0.12 Å/s respectively by heating them at temperatures of around 300 and 130°C respectively.

The n-C_{60} layer used in the nnp RJ was obtained by co-subliming C_{60} and PhIm at 0.5 and 0.2 Å/s respectively at a temperature of around 400 and 130°C respectively.

For the pure charge transport molecules (TaTm and C_{60}), a temperature of around 250 °C was used to maintain a rate ranging from 0.2 to 0.3 Å/s.

For the perovskite deposition, all substrates were transferred to a different vacuum chamber equipped with the same setup described above but destined only for perovskite deposition to avoid cross-contamination. Three crucibles were introduced in the chamber containing the

three perovskite precursors: CsI, FAI, and the $Pb(I_{1-n}Br_n)_2$ mix halide precursor. The latter was pre-synthesized by mixing PbI_2 and $PbBr_2$ powder in a weight ratio of 8:1 (PbI_2:$PbBr_2$) and heating it up to 360°C in N_2 at atmospheric pressure. The CsI, FAI and $Pb(I_{1-n}Br_n)_2$ were heated to around 400, 150, and 250°C respectively to achieve rates of 0.09, 0.45, and 0.45 Å/s respectively. The rates were monitored using QCM sensors placed above each source.

ITO was deposited via Pulsed Laser Deposition using a *Solmates large area PLD 200 mm system.* The PLD tool is coupled to an N2-filled glovebox, avoiding the negative impact of O2 and moisture on the fabricated devices. A *Lightmachinery's IPEX-700 KrF excimer laser* (ʎ = 248 nm) is used. The repetition rate is set at 25 Hz and the fluence at 1.5-1.6 J cm^{-2}. A SnO_2:In_2O_3 ceramic target with 2:98 wt.% from Pi-kem is employed as source material for the deposition of ITO. The overall chamber pressure during the deposition process is set at 0.033 mBar while the oxygen partial pressure (P_{O2}) is fixed at 0.0075 mBar which accounts for an O_2:Ar ratio of 1:4. To achieve the desired thicknesses of 20 nm and 5 nm for the ITO RJs and of 80 nm for the top electrode, the recipe was runn for a total of 7, 2 and 25 cycles.

SnO_2 and Al_2O_3 are deposited via ALD using an Arradiance's GEMStar XT Thermal ALD system integrated into an N_2-filled glovebox. For SnO_2 deposition, the ALD chamber is heated to 60°C, the bottle containing the Sn precursor tetrakis(dimethylamino)tin (TDAT) was heated to 60°C, and the bottle of water (oxidizer) was not heated. The precursor manifolds were heated to 115 and 140°C respectively. The SnO_2 deposition process consists of a series of purges of TDAT for 550 ms and water vapor for 200 ms, each of them followed by N_2 purges to clear off the precursors from the ALD chamber. The ALD cycle for Al_2O_3

deposition consists of consecutive purges of TMA for 10 ms and water for 30 ms followed each by N_2 purges to remove the precursors from the ALD chamber. The chamber is heated to 40°C, and the TMA precursor and water oxidizer are heated to 115 and 140°C respectively.

Silicon bottom cell

The bottom cell is a rear-junction, SHJ cell which comprises the following layers from top to bottom: an n-type hydrogenated amorphous silicon layer (a-Si:H (n)), an intrinsic hydrogenated amorphous silicon layer (a-Si:H (i)), an n-doped crystalline silicon absorber (n-type c-Si), an a-Si:H (i) layer, a p-type hydrogenated amorphous silicon layer (a-Si:H (p)) which forms the rear junction with the n-typ c-Si absorber, and finally a layer of ITO and Ag as bottom electrode.

General Characterization

The current density-voltage (J-V) characteristics were obtained using a Keithley 2612A SourceMeter. The devices were illuminated through shadow masks using a Wavelabs Sinus 70 LED solar simulator.

Hall Measurement of n-C_{60}

We deposited 150 nm of n-doped C_{60} following the same recipe as previously detailed. The measurement was performed at room temperature (30 ˚C) and at a working pressure below 8×10−4 mbar using a thin film analyzer (TFA) from Linseis. The result is shown in Figure S4.

As stated in the manuscript, the presence of artifacts during EQE measurements has been observed previously. Here, we show how heavily shunted cells can display even more

dramatic results if no electrical biasing is applied. In Figure S.1, we see that using our organic junction, the influence of biasing does not produce significant effects on the measurements (left Figure). However, the sample with 5 nm of ITO (right Figure) shows an interesting behavior: while biasing with infrared light, the only way to measure the top cell is by using the right bias (positive V_{OC} of the silicon cell). Any other voltage results in a partial measurement of the top and bottom cells (0V). When biasing with blue light, we observed less dramatic effects of shunts (lower signal in the infrared, higher in the visible). To partially understand these results, one would need to consider not only the shunts (in both sub-cells) but also the reverse breakdown voltage. To our knowledge, a full description of this kind of behavior has not been provided.

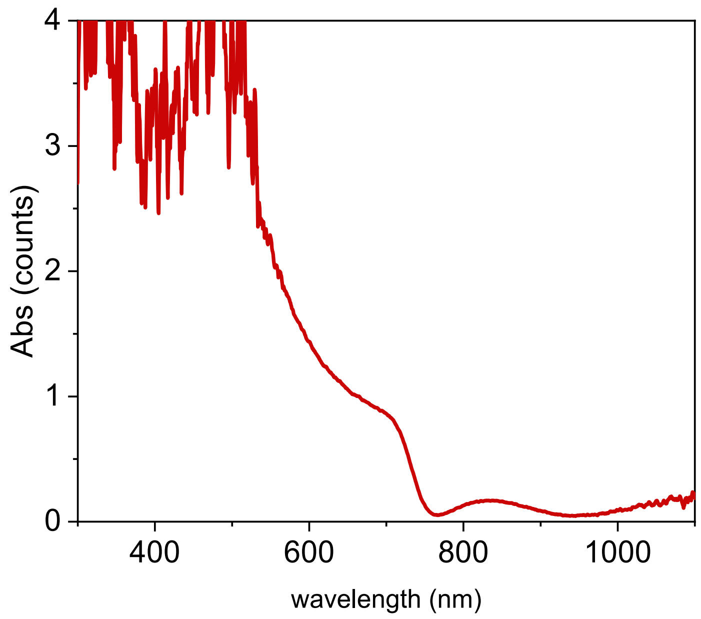

Figure S1: Absorbance spectra of our $Cs_{0.2}FA_{0.8}Pb(I_{0.8}Br_{0.2})_3$ wide band gap perovskite film.

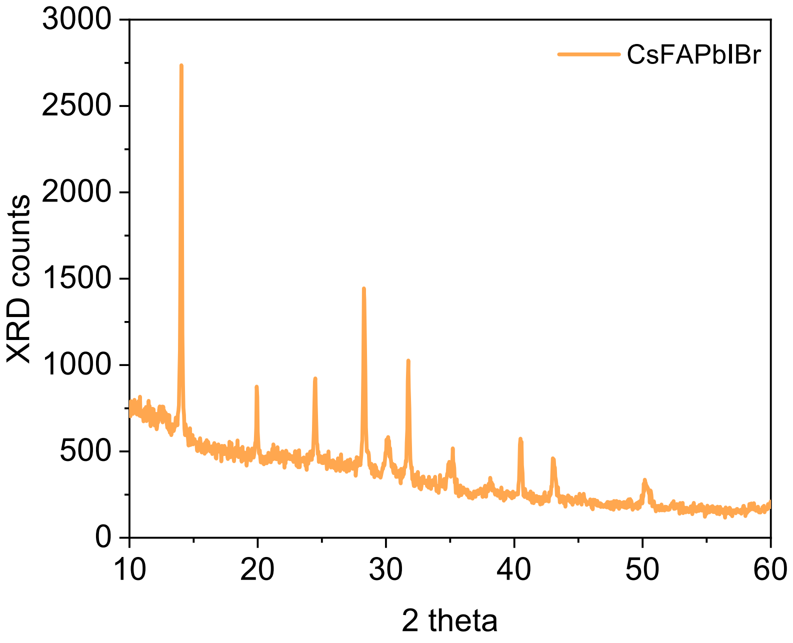

Figure S2: XRD spectra of wide band gap $Cs_{0.2}FA_{0.8}Pb(I_{0.8}Br_{0.2})_3$ perovskite used to make the top layer of our tandem device.

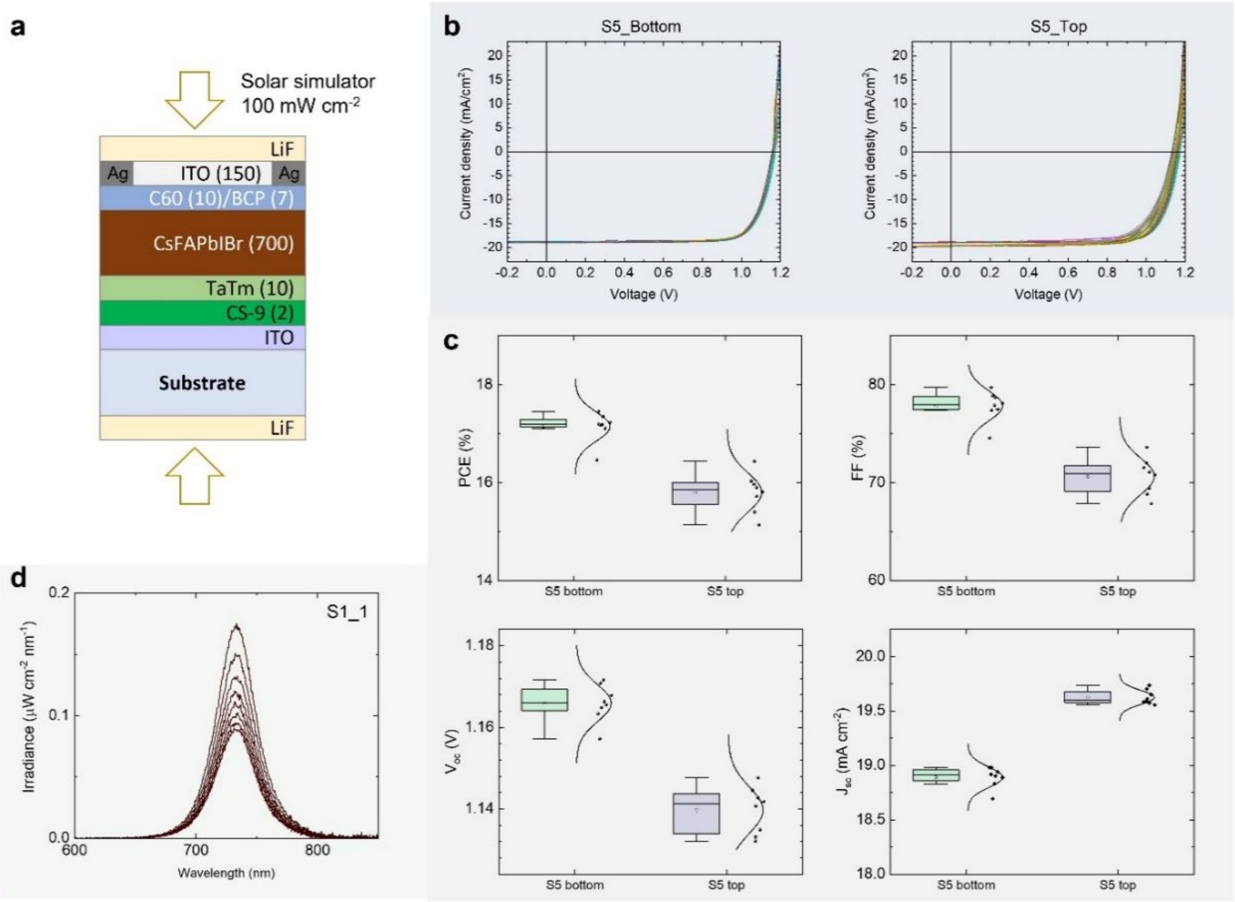

Figure S3: Reference single junction wide band gap $Cs_{0.2}FA_{0.8}Pb(I_{0.8}Br_{0.2})_3$ semitransparent solar cell device statistics, measured from both top and bottom illumination.

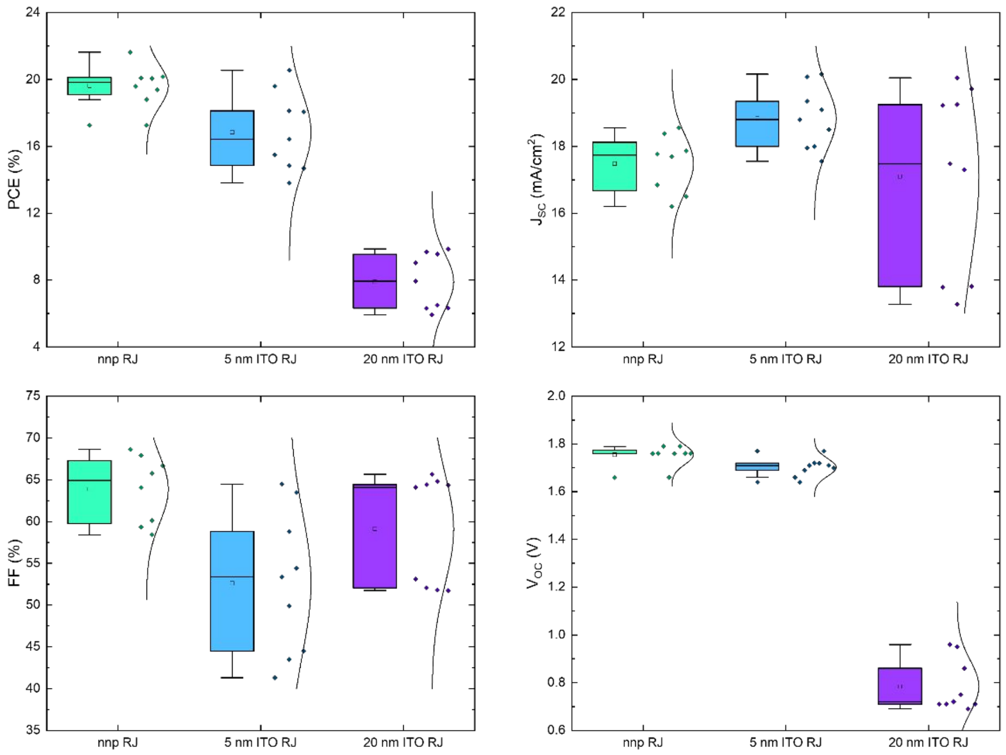

Figure S4: Statistics of the key performance parameters for the tandem cells with the three different RJ's as obtained from J-V scans under simulated sunlight.

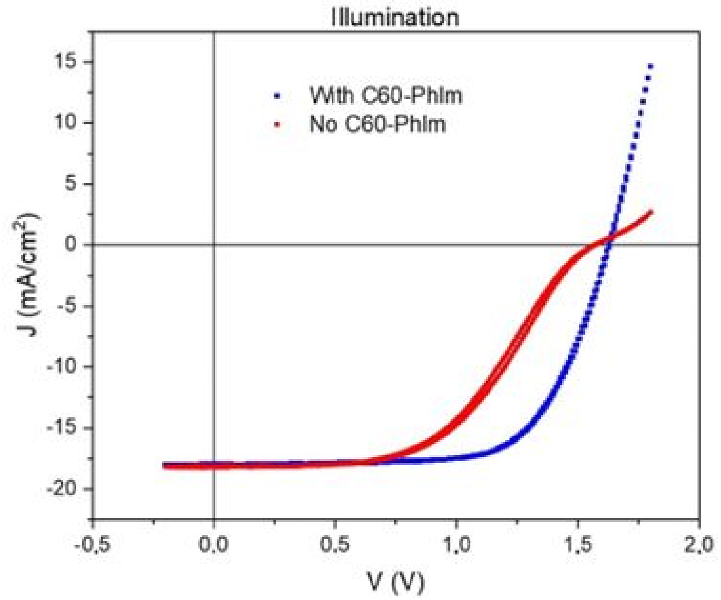

Figure S5: JV characteristics under illumination of a tandem device with the p-doped organic conjugated arylamine (TaTm) hole transport layer of the top perovskite cell directly on top of the n-doped hydrogenated amorphous silicon of the bottom silicon cell (red curve) and of a tandem device featuring our organic n-n-p RJ (blue curve)

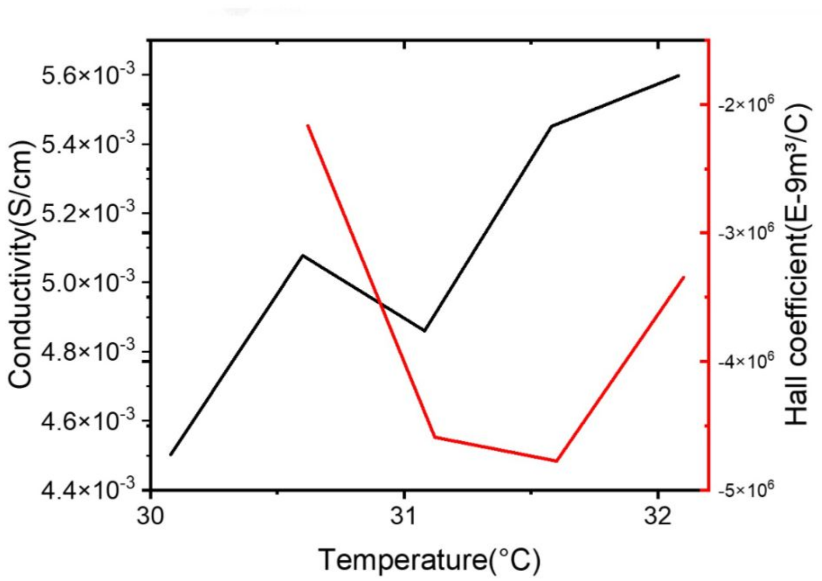

Figure S6: Hall effect measurements of 150nm of doped C_{60}. The negative value of the Hall coefficient denotes the n type semiconductor type.

As can be seen in Figure S6, the measured Hall coefficient has negative values indicating an n-type semiconductor nature. It must be taken into account that the conductivity of the layer is very close to the detection limit of our setup, which explains the variability in the Hall coefficient. However, the conductivity value obtained is in agreement with the one used in the manuscript.

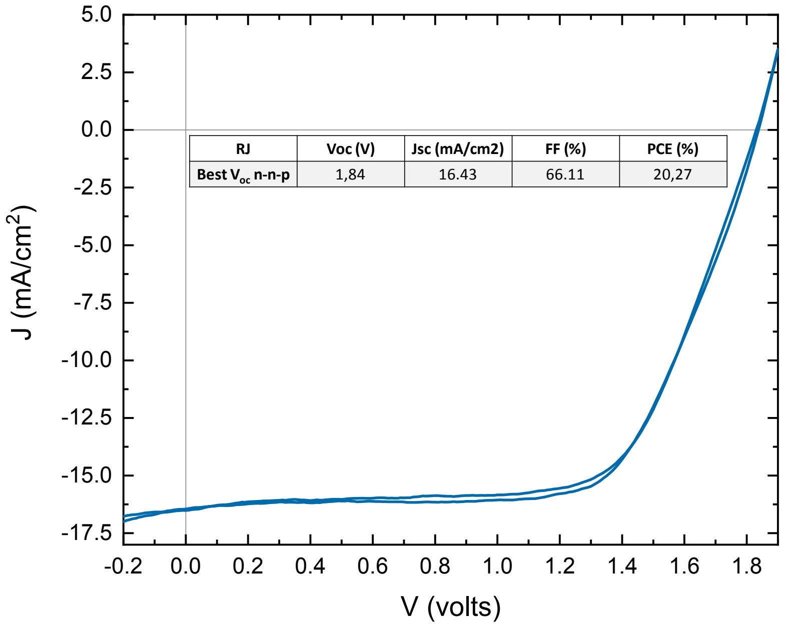

Figure S7: JV characteristics under illumination of the tandem cell comprising our organic n-n-p RJ with the highest achieved voltage to date.

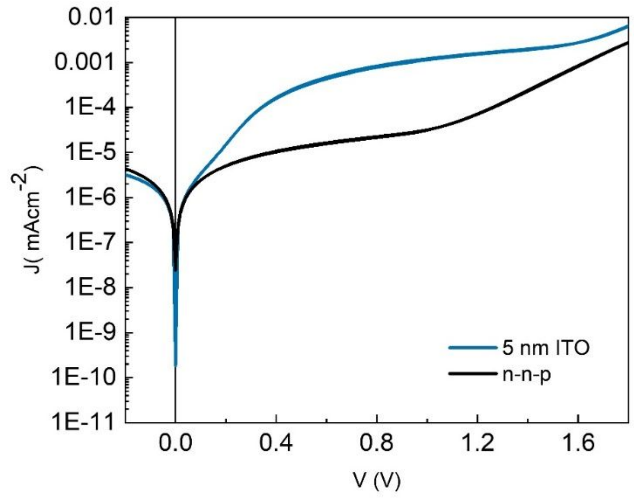

Figure S8: Dark JV characteristics of a tandem device featuring a 5 nm thick RJ (blue curve) and a tandem device featuring our organic n-n-p junction (black curve). Again, the presence of shunts in the ITO sample is evident by the large amount of leakage current.

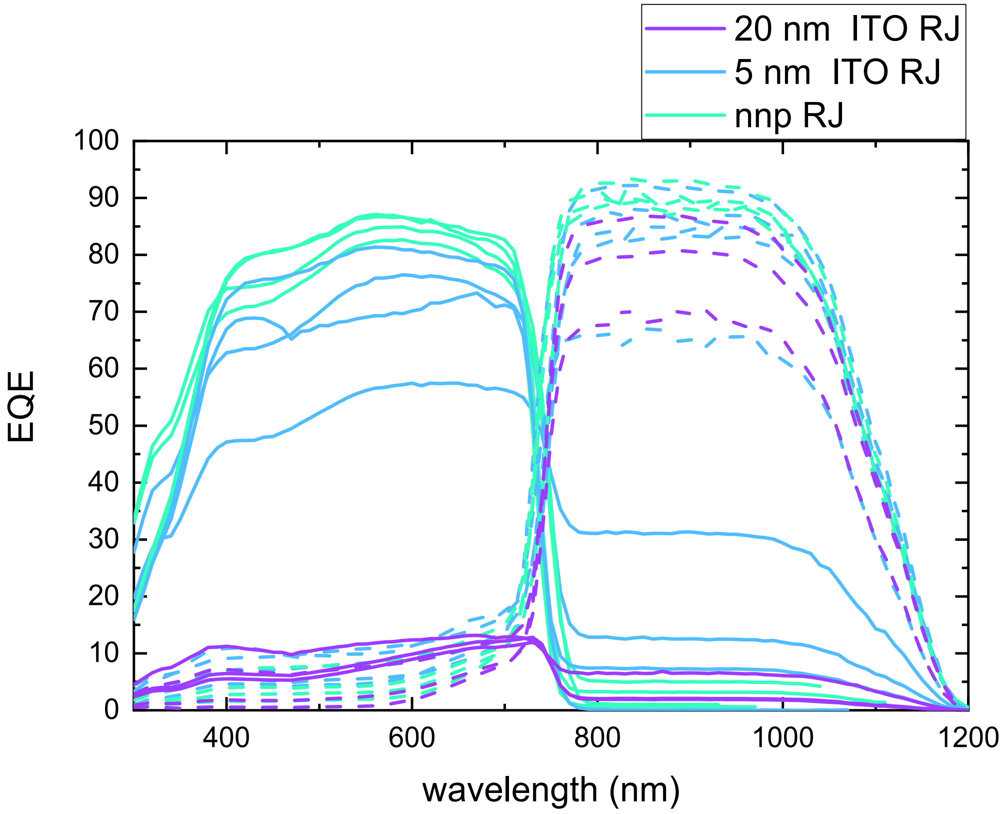

Figure S9: Statistics of the EQE data for the three types of tandem cells.

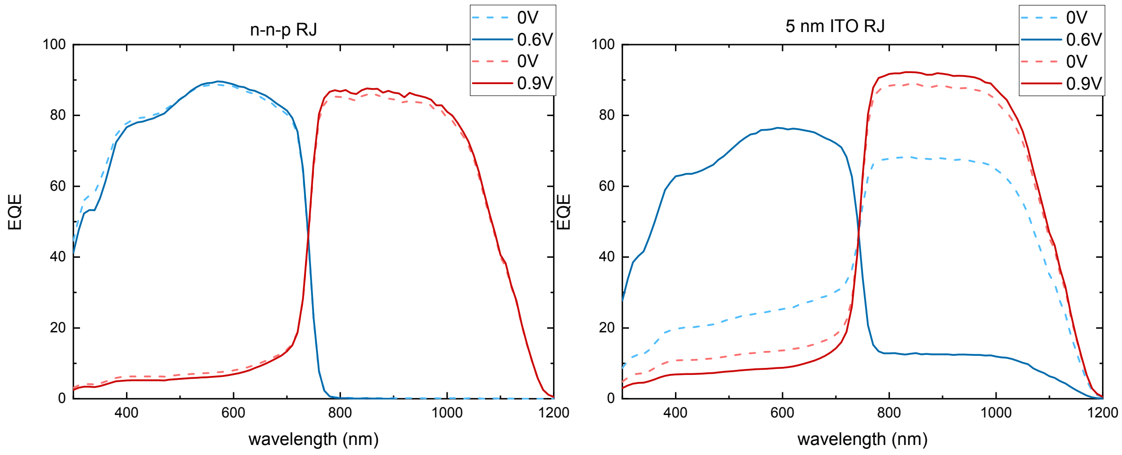

Figure S10 EQE under different voltage biases for a) Tandem device with the organic n-n-p RJ proposed in this work and b) Tandem device with a 5 nm thick ITO RJ.

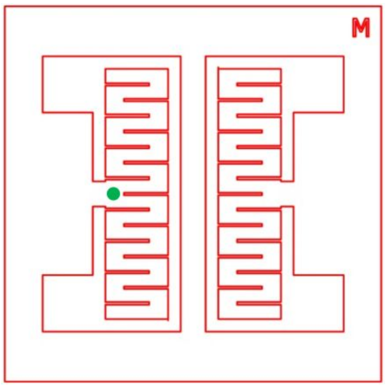

Figure S11: Diagram of the metal fingers shadowing 11% of the active area of our tandem device. In green it can be seen where we position the probe beam of the QE-R Enlitech EQE setup.

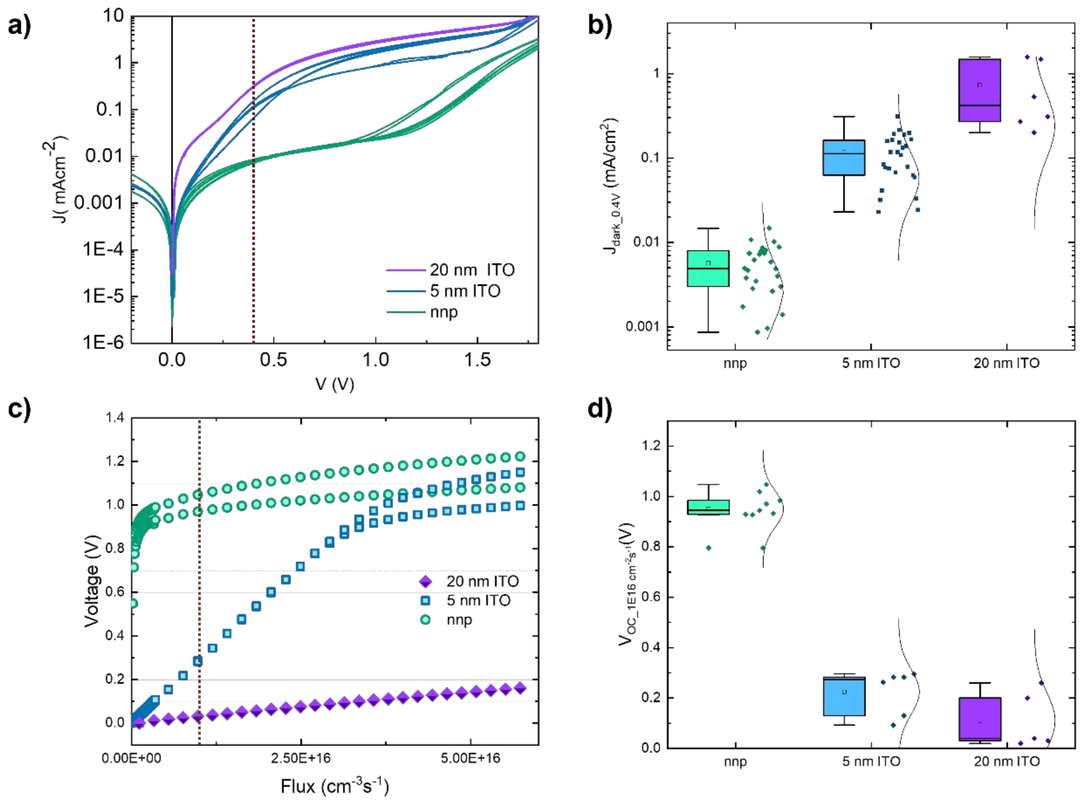

Figure S12 (a) Dark JV curves for one deposition run of tandem devices comprising 20 nm ITO RJ (violet), 5nm ITO RJ (blue) and n-n-p RJ (green). (b) Statistics for over 20 devices for 5nm ITO RJ and nnp RJ, and 5 samples for 20nm ITO RJ of the dark current density measured at 0.4V (c) Suns-V_{OC} technique measured with blue 450nm laser to measure the voltage in the perovskite sub-cell for both RJ types, 20 nm ITO RJ (violet), 5nm ITO RJ (blue) and n-n-p RJ (green). (d) Statistics for over 20 devices for 5nm ITO RJ and nnp RJ, and 5 samples for 20nm ITO RJ of the value of V_{OC} at a flux of $1x10^{16}$ (cm^{-2}s^{-1}).

Device Statistics

Herein, we present data of different cells with the same architecture as the data presented in the manuscript, for both 5nm of ITO (blue curves) and of nnp RJ (green curve) for over

20 different tandem cells in the cases of 5 nm ITO RJ and nnp RJ, while fewer samples (5) for 20 nm ITO (violet curve) due to the very low efficiency obtained in each batch. The effect of the shunts is seen clearly from the dark JV curves (Figure S12 a) and Suns-V_{OC} while illuminating with the blue laser (Figure S12 c). In the dark J-V curves the low voltage region is the one associated with leakage currents, with lower current values indicating that the shunts have less impact (Figure S12 b). For the Suns- V_{OC} with selective illumination analysis, the voltage vs Flux curve of the silicon bottom cell is omitted to enhance the clarity since we aim to compare only the effect of the recombination junction on the shunts of the top cell. A tandem device without shunts in the top cell would follow a logarithmic increase in the voltage vs flux when illumination with the 450 nm laser, whereas a (partially) shunted top cell would show a more linear increase in the voltage as a function of flux (Figure S12 c). As can be seen in Figures S12 b and d, the over 20 devices compared here show a similar behavior, in which samples composed of the n-n-p RJ show higher shunting resistance.
